# Numerical optimization of pacing strategy in cross-country skiing based on Gauss pseudo-spectral method

**DOI:** 10.1038/s41598-022-24859-2

**Published:** 2022-11-28

**Authors:** Xu Ni, Jiawei Liu, Shuguang Zhang, Peng Ke

**Affiliations:** School of Transportation Science and Engineering, Beihang University, Beijing, 100191 China

**Keywords:** Biomedical engineering, Mechanical engineering

## Abstract

In cross-country skiing competitions, the choice of pacing strategy is of decisive significance to athletes' performance. A reasonable pacing strategy is essential for athletes to improve their performance. In this paper, the mathematical models of cross-country skiing simulation and pacing optimization are established, including motion model, athlete power output model, and optimization model. The actual competition data of a Chinese athlete in Guyangshu 1.5 km track was compared with the model simulation results. The whole process time error is less than 3%, which verifies the accuracy of the motion and power output model. Gauss pseudo-spectral method is applied to the optimization model. By changing the distribution of athletes' power output, the racing time is minimized under the condition that the total energy output remains unchanged. Compared with the pacing strategy before optimization, the optimized racing time was shortened by 12.6 s, which verifies the effectiveness of the optimization model. Optimized results show that in the first significant uphill section, a recommendation is to use a more conservative strategy, while in the latter half of uphill sections the power output should be increased.

## Introduction

The holding of the Winter Olympics (such as the 2022 Beijing Winter Olympics) has made cross-country skiing and other projects more popular. In order to improve the performance of athletes, in addition to implementing systematic training, it is also very important to conduct scientific research. As stated by Sundström et al., “Mathematical modeling of a ski race, combined with optimization routines, provides a tool for analyzing how these efforts should be best distributed”^1^. Applying optimization theory methods to study the pacing strategy in cross-country skiing can effectively help athletes formulate better training plans and competition strategies to achieve better competition results.

Pacing strategy refers to the athletes’ distribution of energy output during a competition. This concept is often used in endurance sports such as cross-country skiing, marathon, and bike racing. It is of great benefit to studying the pacing strategy of athletes in the competitive process for the breakthrough of performance. Abbiss and Laursen^2^ summarized the durations and race characteristics in general for endurance sports. The results show that in short-distance or long-distance competitions with different rounds and laps, the pacing strategy types mainly include negative, all-out, positive, even, parabolic-shaped and variable pacing strategies.

Swain^3^ in the numerical simulation of cyclic track test shows that the variable pacing strategy is beneficial under varying terrain and ambient wind conditions by adjusting the power output based on the varying terrain and wind conditions. In the simulation model of Underwood and Jeremy^4^, the mechanical characteristics of track banking angle are considered, so the variable power distribution is synchronized with track banking angle.

Dahmen and Saupe^5^ used the previous optimal control algorithm and simulation model to optimize the pacing strategy on the fictitious 2000 m track profile in the road cycling. He evaluated three different bioenergy constraint models, including a three-parameter critical power model and a more realistic six-parameter model. The results show that the power model can better characterize the speed change of the moving process.

In 2013, Sundström et al. also studied cross-country skiing and road cycling and discussed the impact of different slope tracks on the optimal pacing strategy of road cycling and cross-country skiing. Sundström 's research results show that pacing strategies should vary with the slope of the race track. When the external conditions change, the variable pace strategy can obtain a lot of time gain. In 2014, John F. Moxnes^6^ used a power balance model to simulate cross-country skiing in different terrain conditions. They constructed a function of the skier's power output and pacing strategy.

This paper aims to model the motion process of cross-country skiing and study the optimization method of pacing strategy. The optimization algorithm based on Gauss pseudo-spectral method is used to optimize the speed distribution problem of cross-country skiing in the actual scene, give suggestions on pacing strategy, verify the feasibility of pacing strategy, put forward the recommendations of pacing strategy in line with the personal ability of athletes, hoping to improve the actual race performance of athletes.

## Cross-country skiing model

### Skiing track model

**Figure 1:**
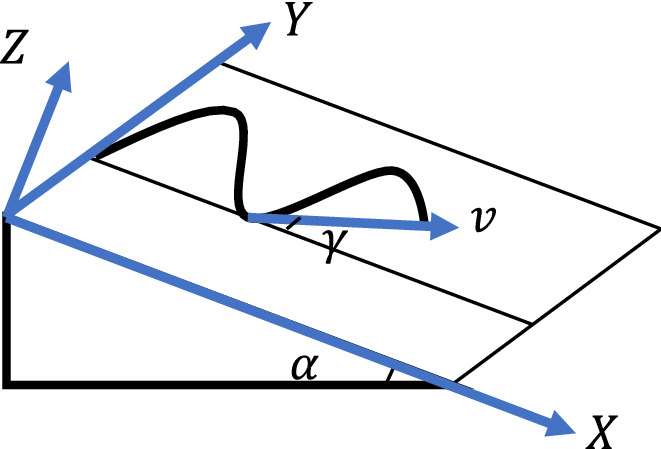
Schematic diagram of track coordinate system.

The race track in cross-country skiing typically consists of terrain both straight, turning, and of varying gradient. This study uses the track surface as the reference system to determine the trajectory and establish the dynamic model, as shown in Fig. 1. Select the direction with the most significant descending gradient along the slope as the positive direction of the x-axis and the direction perpendicular to the upward direction of the track surface as the positive direction of the z-axis to establish the track coordinate system.

The track terrain of cross-country skiing is complex. In most cases, several groups of discrete data points distributed on the track surface represented by $\left(x,y,z\right)$ coordinates are obtained by field measurement or satellite map. These measured discrete points are often irregular. It is difficult to describe the complex surface of the track with a specific function form such as $f\left(x,y\right)$ due to some difficulties in data fitting, so this paper uses the Kriging method, a more agile and accurate grid interpolation method, to reconstruct the track using three-dimensional surface interpolation.

**Figure 2:**
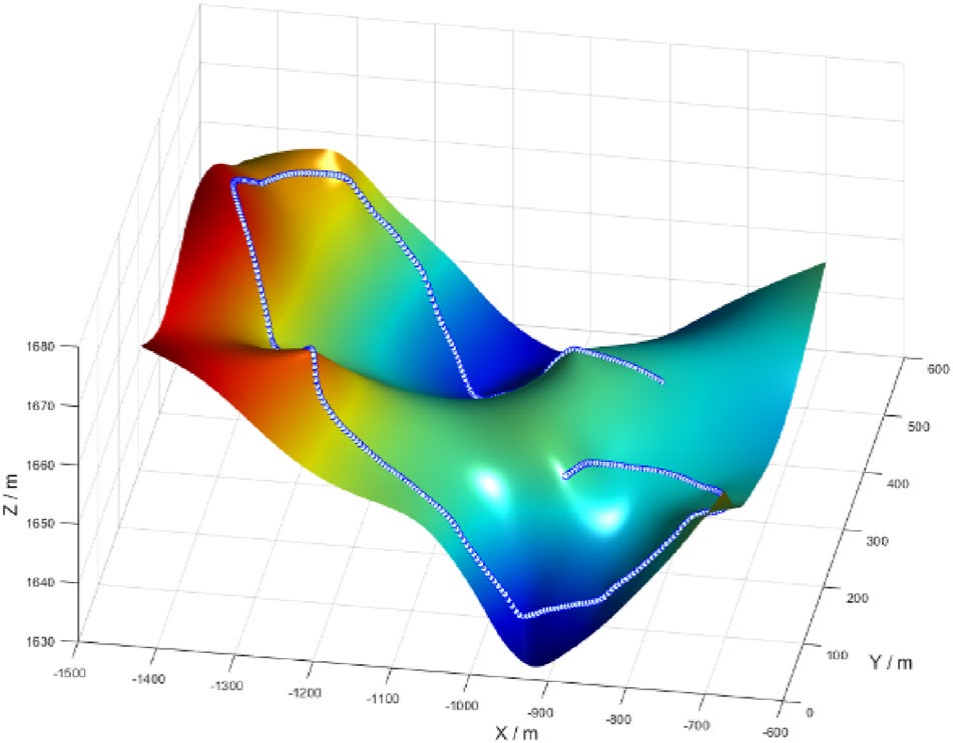
Reconstruction of Guyangshu 1.5 km track.

This paper selects the Guyangshu 1.5 km track in the cross-country skiing competition venue of the 2022 Beijing Winter Olympics for analysis. This cross-country skiing venue is a national cross-country skiing center located in the valley on the southeast side of Zhangjiakou, Hebei Province, China. Aiming at the Guyangshu 1.5 km track, as shown in Fig. 2, a three-dimensional topographic map meeting the path constraints is constructed by extracting the track layout and section map data.

### Athletes driving power model

Hausken^7^ modeled the power output of athletes based on Bolger et al.^8^ 's research on speed and heart rate, expounded the relationship between power output and speed and verified the model with the simulated speed, and actual speed of an elite male skier on the 4218 m track. Based on this model, this paper selects three influencing factors of peak physical fitness to improve and reflect them in pacing strategy optimization.

The instantaneous power of skiers depends on many factors, and the most influential factors are terrain slope and athletes’ speed.

Terrain slope $\alpha =\alpha (t)$ affects power output. Set the slope angle of downhill is negative. In the process of gradually increasing and changing from downhill to uphill, the power gradually increases from 0 and finally stabilizes at the peak power ${P}_{th}$, then the relationship between power and slope can be expressed as an S-shaped curve

1 $$\begin{array}{l}\frac{\partial P}{\partial \mathrm{\alpha }}=bP\left(1-\frac{P}{{P}_{th}}\right)\end{array}$$

The S-curve is often used to simulate the quantity growth of $a$ population. Use slope α to analogize time, $b$ to compare the growth rate of quantity over time, ${P}_{th}$ is similar to the carrying capacity, so the population growth law is similar to the physical model as the slope α. The driving power $P$ increases with the growth rate $b$ until it reaches the skiers critical driving power ${P}_{th}$.

Skiing speed also affects power output. With the decrease of athletes' speed, the power gradually increases and stabilizes at the peak power ${P}_{th}$, the relationship between power and speed can also be expressed as

2 $$\begin{array}{l}\frac{\partial P}{\partial v}=aP\left(\frac{P}{{P}_{th}-1}\right)\end{array}$$

Similar to Eq. (1), parameter $v$ analogizes time, $a$ can also be expressed as the rate at which driving power $P$ decreases with the increase of speed $v$.

Equations (1) and (2), as a function of power and slope ${\alpha }$, and a function of power and speed $v$, simulate the situation when the power increases and decreases, respectively. Solve Eqs. (1) and (2) to obtain the expression of power output. Where parameter $c$ captures the factors affecting the output power except the speed and slope.

3 $$\begin{array}{l}P= \frac{{P}_{th}}{1+{P}_{th}{e}^{\alpha v-b\alpha -c}}\end{array}$$

### Skiing dynamics model

For cross-country skiing, a long-distance race, this paper uses the particle dynamics model to model the athlete's movement process, but an empirical formula is used for the aerodynamic resistance, which is closely related to the athlete's posture.

According to Newton's second theorem and particle dynamics of theoretical mechanics, the dynamic equation in the sliding track coordinate system can be expressed as

4 $$\begin{array}{l}m\left(\frac{\delta {\varvec{V}}}{\delta t}+{\varvec{\omega}}\times {\varvec{V}}\right)=F\end{array}$$

There are multiple external forces that can be defined in the trajectory coordinate system, and they can be decomposed into aerodynamic resistance ${F}_{D}$, Gravity ${F}_{G}$, friction resistance between snow surface and ski ${F}_{f}$ and ground support force ${F}_{N}$. The gravity and ground support force are defined in the ground coordinate system and ski coordinate system respectively. Therefore, the force projection in the track coordinate system needs to be obtained with the help of the corresponding coordinate system conversion matrix.

The projection formulas of speed, angular velocity, and external forces are brought into Eq. (4), and finally, the scalar form of the athlete's centroid dynamic equations under the track coordinate system is obtained:

5 $$\begin{array}{l}\left\{\begin{array}{l}m\dot{v}={F}_{s}-{F}_{f}-{F}_{D}+mg\left(\mathrm{sin}{\alpha }_{1}\mathrm{cos}\gamma -\mathrm{cos}{\alpha }_{1}\mathrm{sin}{\alpha }_{2}\mathrm{sin}\gamma \right)\\ mv\left(\dot{\gamma }+{\dot{\alpha }}_{1}\mathrm{sin}{\alpha }_{2}\right)=-{F}_{N}\mathrm{sin}\beta -mg\left(\mathrm{sin}{\alpha }_{1}\mathrm{sin}\gamma +\mathrm{cos}{\alpha }_{1}\mathrm{sin}{\alpha }_{2}\mathrm{cos}\gamma \right)\\ mv\left({\dot{\alpha }}_{1}\mathrm{cos}{\alpha }_{2}\mathrm{cos}\gamma +{\dot{\alpha }}_{2}\mathrm{sin}\gamma \right)={F}_{N}\mathrm{cos}\beta -mg\mathrm{cos}{\alpha }_{1}\mathrm{cos}{\alpha }_{2}\end{array}\right.\end{array}$$

where ${F}_{s}$ represents driving force, ${F}_{f}$ represents frictional resistance, ${F}_{D}$ represents aerodynamic resistance, ${F}_{N}$ represents support force, ${\alpha }_{1}$ represents vertical terrain slope angle, ${\alpha }_{2}$ represents lateral terrain slope angle, $\gamma$ represents the angle between speed direction and X axis of track coordinate system, and $\beta$ is the ski tilt angle.

The formula for calculating air resistance is

6 $$\begin{array}{l}{F}_{D}= -\frac{1}{2}\rho {{v}_{f}}^{2}\cdot A{C}_{D}\left({R}_{e}\right)\end{array}$$

The expression of $A$ and ${C}_{D}$ adopts the fitting formula of Losnegard^9^. Firstly, the windward area is normalized by ${m}^\frac{2}{3}$, where $m$ is the body weight and $\frac{2}{3}$ is the allometric scale index; Secondly, the standardized windward area can be assumed to be an S-type function of ski speed $v$^10^, and the logical function about the windward site is obtained:

7 $$\begin{array}{l}\frac{A}{{m}^\frac{2}{3}}= {\zeta }_{1}- \frac{{\zeta }_{2}}{1+{e}^{ -\left(\frac{v}{{\zeta }_{4}} - {\zeta }_{3}\right)}}\end{array}$$

The four-parameter vector $\zeta$ was determined by minimizing the sum of the squared residuals from the normalized estimates of drag area ($\frac{A}{{m}^\frac{2}{3}}$) determined using the Levenberg–Marquardt algorithm. Hence, once $\zeta$ was established, the only necessary input was body mass and skiing speed.

Air drag coefficient is expressed as a function of Reynolds number. The S-shaped curve is used to fit the data. Specifically, the four-parameter vector η was determined by minimizing the sum of the squared residuals between measurements and the model in Eq. (8) using the Levenberg–Marquardt algorithm:

8 $$\begin{array}{l}{C}_{D}\left(Re\right)= {\eta }_{1}- \frac{{\eta }_{2}}{1+ {e}^{-\left(\frac{Re}{{\eta }_{4}}- {\eta }_{3}\right)}}\end{array}$$

The specific definition and solution of parameters $\zeta$ and $\eta$ can be found in^10^.

The formula for calculating friction resistance is

9 $$\begin{array}{l}{F}_{f}=\frac{\mu \left({v}^{2}+g{R}_{xz}\mathit{cos}{\alpha }_{1}\right)}{{R}_{xz}}\end{array}$$

where ${R}_{xz}$ represents the radius of curvature generated when the slope fluctuates during the athletes' progress in Fig. 1, and $v$ is the windward speed, $\mu$ is the dynamic friction coefficient between ski board and snow.

To determine the sliding track of athletes on the ground, it is necessary to establish the kinematic model of athletes in the sliding process. In this process, the dynamic equations need to be solved. The fourth-fifth order Runge Kutta algorithm solves the nonrigid ordinary differential equations^11^. Input the initial value of each variable, solve the differential equation by using the characteristic of adaptive step size of this method, and obtain the dynamic parameters of the athlete during skiing. We select the skiing speed and project it into the ground coordinate system by using the transformation matrix to get the kinematics model in scalar form as follows:

10 $$\begin{array}{l}\left\{\begin{array}{l}\frac{dx}{dt}=v\left(\mathrm{cos}{\alpha }_{1}\mathrm{cos}\gamma +\mathrm{sin}\gamma \mathrm{sin}{\alpha }_{1}\mathrm{sin}{\alpha }_{2}\right)\\ \frac{dy}{dt}=v\mathrm{cos}{\alpha }_{2}\mathrm{sin}\gamma \\ \frac{dz}{dt}=v\left(-\mathrm{sin}{\alpha }_{1}\mathrm{cos}\gamma +\mathrm{sin}\gamma \mathrm{cos}{\alpha }_{1}\mathrm{sin}{\alpha }_{2}\right)\end{array}\right.\end{array}$$

### Model verification

**Figure 3:**
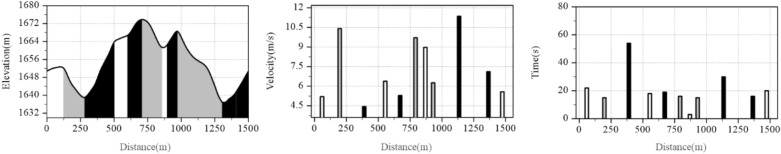
Terrain of Guyangshu track, velocity and time distribution of athlete A.

Taking the race process of a Chinese athlete A at the Guyangshu 1.5 km track as an example, the actual race data of athlete A at the Guyangshu track are compared with the simulation results based on the cross-country skiing model to verify the accuracy of the cross-country skiing model. Figure 3 shows the terrain of Guyangshu track and the actual race data of athlete A.

**Figure 4:**
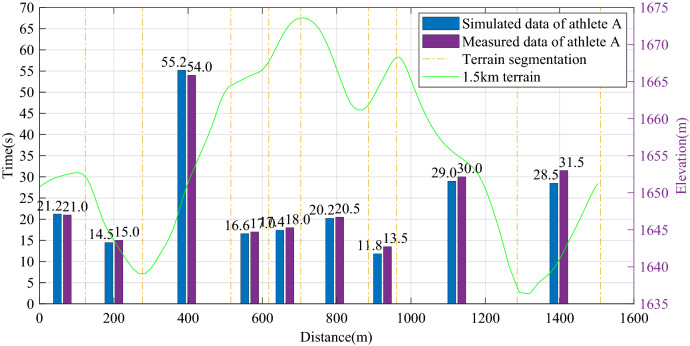
Comparison of athlete A’s measured data and simulation data.

According to the relationship between maximum oxygen uptake and peak power proposed by Burnley and Vanhatalo^12^, and using the track test results of athlete A, the athlete's power curve is calculated, as shown in Fig. 4.

The Guyangshu 1.5 km track is divided into nine segments. By comparing the measured time of each segment and the simulation time of the model, the cross-country skiing model can accurately predict the movement time of each segment, the maximum error is no more than 3 s, and the percentage of the whole process time error is 2.77%. Therefore, the model has good accuracy.

Through the mathematical modeling of cross-country skiing track, power output, and race process, the cross-country skiing model established the functional mapping relationship between terrain, physical parameters, and competition time, accurately predicting the movement process of each segment of cross-country skiing verified the model. As the universal calculation framework of the cross-country skiing process, the model can adjust the terrain and physical parameters according to the different venues and athletes to obtain accurate simulation results, laying a foundation for the pace optimization process.

### Republication of results

The method proposed in this paper can be implemented on a similar model by following the flowchart illustrated in Fig. 6. The design problems, dynamic model, and boundary conditions are given in this paper. If the information provided in the paper is not enough, we sincerely welcome scientists or interested parties to contact us for further explanation.

## Pacing strategy optimization method

### Objectives and constraints

Pacing strategy optimization refers to finding the best speed change mode in the race process. In addition, the pacing strategy optimization in endurance sports is usually used to improve the performance, which can be expressed in the completion time, so the objective function is the duration between the beginning and the end. In addition, certain restrictions must be applied so that the solution does not exceed reasonable limits. These mathematical constraints depend on the number of solutions in the bioenergy model and the equation of motion.

The objective function or performance index is expressed as

11 $$\begin{array}{l}T= \sum_{i=1}^{K}\Delta {t}_{i}\end{array}$$

Besides the dynamic constraints in Eqs. (5) and (10), and the terrain constraint in “Model verification”, the energy and power output constraint are confined and expressed as

12 $$\begin{array}{l}\left\{\begin{array}{l}{\sum }_{k=1}^{K}{E}_{k}\le {E}_{total}\\ 0\le \frac{{P}_{th}}{1+{P}_{th}{e}^{\alpha v-b\alpha -c}}\le {P}_{max}\end{array}\right.\end{array}$$

Therefore, the pacing strategy optimization problem minimizes the completion time by changing the distribution curve of power output along the track to meet the terrain position constraints, total energy constraints, explosive force constraints, and dynamic constraints. Due to the existence of reasonable constraints, the results of pacing strategy optimization do not put forward higher requirements for athletes' explosive power and endurance but change the distribution of power output over time. Therefore, athletes can quickly adapt to the optimized new pacing strategy and apply it to the competition to achieve better results.

### Optimization method

Gauss pseudo-spectral method is used in the process of pace optimization. On the premise of meeting the optimization constraints, athletes’ power output in each segment is changed to achieve shorter exercise time.

The optimal control problem composed of the initial continuous-time domain system and the performance function to be optimized can be discretized by Gauss pseudo-spectral method and approximated by global polynomial interpolation. Finally, it can be transformed into solving the NLP problem. The condition of the NLP problem is equivalent to the first-order necessary condition of the discrete form of the optimization problem. This means that from the mathematical principle, the solution calculated by the NLP problem is equivalent to the optimal solution of the original optimal control problem^13^,^14^, which is comprehensively expressed as follows.

13 $$\begin{array}{l}\left\{\begin{array}{l}\underset{{U}_{i}\mathit{ },i=\mathrm{1,2},\dots ,N}{\mathrm{min}} \left\{J= \varphi \left({X}_{0},{t}_{0},{X}_{f},{t}_{f}\right)+\frac{{t}_{f}-{t}_{0} }{2}{\sum }_{k=1}^{N}{\widehat{w}}_{k}g\left({X}_{k},{U}_{k},{\tau }_{k}{;t}_{0},{t}_{f}\right) \right\}\\ \mathrm{s}.\mathrm{t}. \,\, \sum_{i=0}^{N}{D}_{k,i}\left(\tau \right){X}_{k}=\frac{{t}_{f}-{t}_{0}}{2}f\left({X}_{k},{U}_{k},{\tau }_{k}{;t}_{0},{t}_{f}\right), \quad K={1,2,} \ldots {\text{N}}\\ B\left({X}_{0},{t}_{0},{X}_{f},{t}_{f}\right)=0\\ C\left({X}_{k},{U}_{k},{\tau }_{k}{;t}_{0},{t}_{f}\right)\le {0}, K={1,2,} \ldots {\text{N}}\\ {X}_{f}- {X}_{0}- \sum_{i=0}^{N}{X}_{i}\sum_{n=1}^{N}{w}_{k}{D}_{ni}=0\end{array}\right.\end{array}$$

where $\dot{x}(t)=f(x(t),u(t),t)$, $B\left(x\left({t}_{0}\right),{t}_{0},x\left({t}_{f}\right),{t}_{f}\right)=0$, $C\left(x\left(t\right),u\left(t\right),t\right)\le 0$, represent general nonlinear system state equation, boundary conditions and path constraints; $x(t)$ is the state variable, and $u\left(t\right)$ is the control variable; $J$ represents performance index function; $t\in \left[{t}_{0},{t}_{f}\right]$ is the time range; ${D}_{k,i}$ is an N × (N + 1) state differential matrix, which can be calculated in advance; and ${w}_{k}$ is Gauss integral weight.

**Figure 5:**
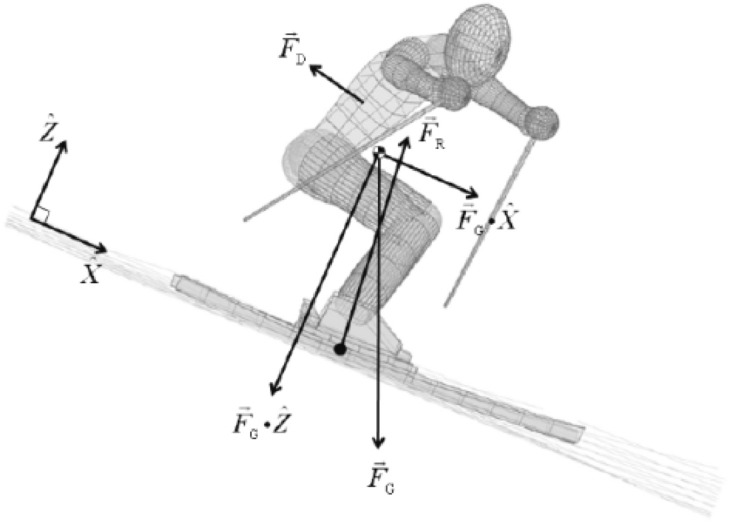
Force analysis diagram^15^.

In solving the NLP problem, the covariate variables in the optimal control problem can be accurately evaluated through the necessary conditions for the optimal solution of the nonlinear programming problem (Karush Kuhn Tucker, KKT). As shown in Fig. 5^15^, when the initial conditions are known, the dynamic trajectory planning problem can be transformed into a series of static optimization problems^13,16^; in fact, this process is to transform the two-point boundary value problem corresponding to the continuous first-order necessary condition of the original optimization problem into an initial value problem^17^.

Gauss pseudo-spectral method transforms the trajectory optimization problem in the continuous-time domain into a discrete trajectory planning problem. It then uses standard value algorithms such as sequential quadratic programming (SQP) and interior point method (IPOPT) to solve it.

**Figure 6:**
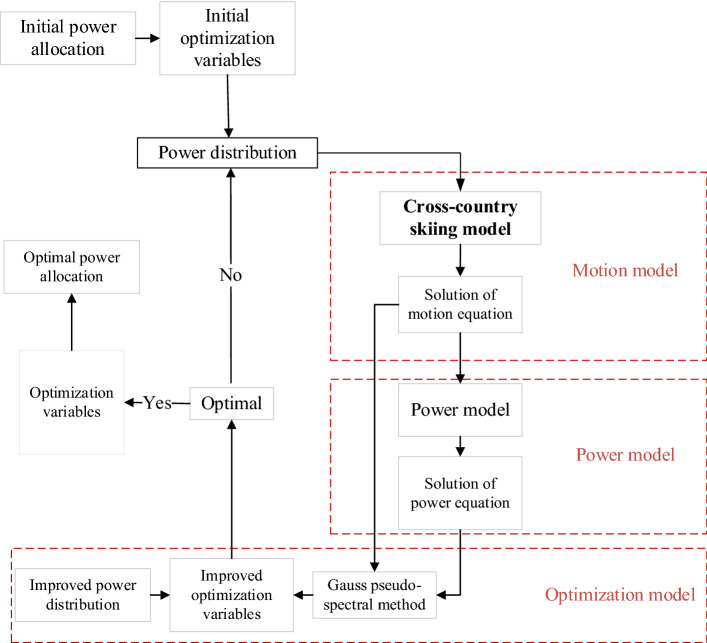
Pacing strategy optimization flow chart^15^.

The pacing strategy optimization model based on Gauss pseudo-spectral method is shown in Fig. 6.

Since adding three-dimensional constraints when solving the optimization problem by Gauss pseudo-spectral method is challenging, the cross-country skiing route is simplified to a certain extent. The three-dimensional skiing track is stretched into a two-dimensional curve in the plane composed of forwarding direction and altitude direction. Without considering the turning process in the skiing track, the expressions of dynamic and kinematic constraints in the speed optimization model can be simplified.

14 $$\begin{array}{l}\left\{\begin{array}{l}\frac{dv}{dt}= \frac{\frac{P}{v}}{\mathrm{m}}-g\mathit{sin}{\alpha }_{1}-\frac{\frac{1}{2}\rho {C}_{d}A{v}^{2}}{\mathrm{m}}-\frac{\mu \left({v}^{2}+g{R}_{xz}\mathit{cos}{\alpha }_{1}\right)}{{R}_{xz}}\\ \frac{dx}{dt}=v\mathit{cos}{\alpha }_{1}\end{array}\right.\end{array}$$

**Figure 7:**
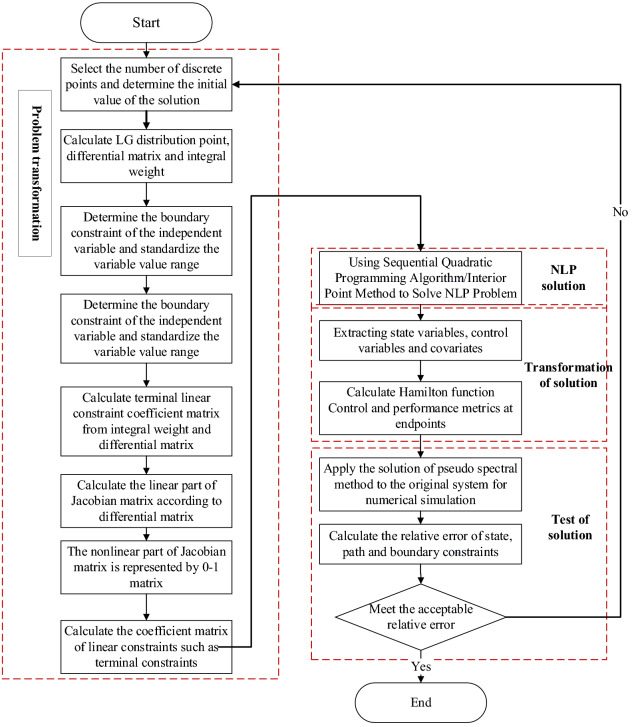
Iterative flow chart of pacing optimization based on Gauss pseudo-spectral method.

The specific solution process of the Gauss pseudo-spectral method in the pacing optimization problem is shown in Fig. 7.

## Pacing strategy optimizations

### Pacing strategy based on athlete A’s data

Taking Chinese athlete A as an example, according to the collected speed time data and physiological data of athlete A, the constraints of time, energy, and power of athlete A's pacing strategy optimization problem can be obtained as follows:

15 $$\begin{array}{l}\left\{\begin{array}{l}\sum_{i=1}^{11}\Delta {t}_{i}\le 216.6 \mathrm{s}\\ {\sum }_{k=1}^{11}{E}_{k}\le 57.2 \mathrm{kJ}\\ 0\le P\le 350 \mathrm{W}\end{array}\right.\end{array}$$

Using the optimization algorithm based on the Gauss pseudo-spectral method, the speed of athlete A in the whole process of Guyangshu 1.5 km track before and after optimization and the corresponding skiing time and speed of each segmentation can be obtained, as shown in Table 1.

**Table 1:** Comparison of race data of athlete A before and after optimization.

Parameters	Before optimization	After optimization
Time (s)	215	203
Maximum speed (m/s)	18.7	18.4
Total output energy (kJ)	57.2	57
Peak power (W)	348.7	349.3
Average power (W)	264.1	279.4

Athletes data used in the calculation process are shown in the appendix. The study was approved by the China Institute of Sports Science. Each subject provided written informed consent prior to taking part in the study and the methods were carried out in accordance with the relevant guidelines and regulations.

**Figure 8:**
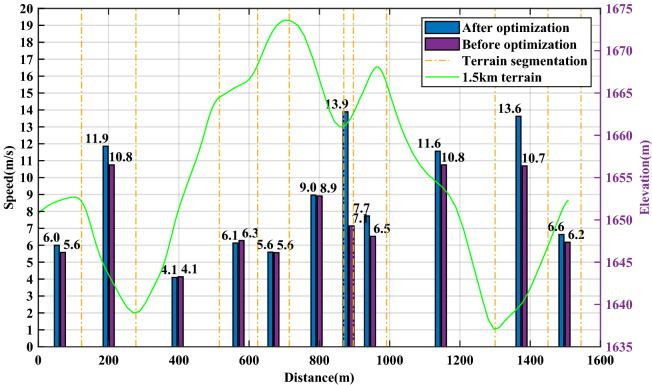
Speed distribution of athlete A before and after optimization.

**Figure 9:**
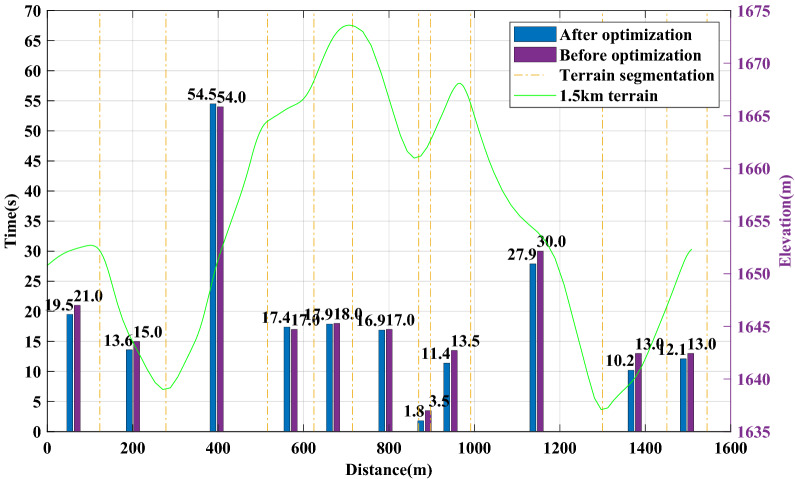
Time distribution of athlete A before and after optimization.

According to the segmented average time and speed distribution shown in Figs. 8 and 9, the gain of performance is mainly in two uphill and one downhill in the second half of the journey, and the time is shortened from 13.5 s to 11.4 s, 30 s to 27.9 s and 13.3 s to 10.2 s, respectively. Therefore, a critical difference between the pacing strategies before and after optimization is that the optimized pacing strategy achieves sufficient acceleration in the initial downhill section of the second half.

**Figure 10:**
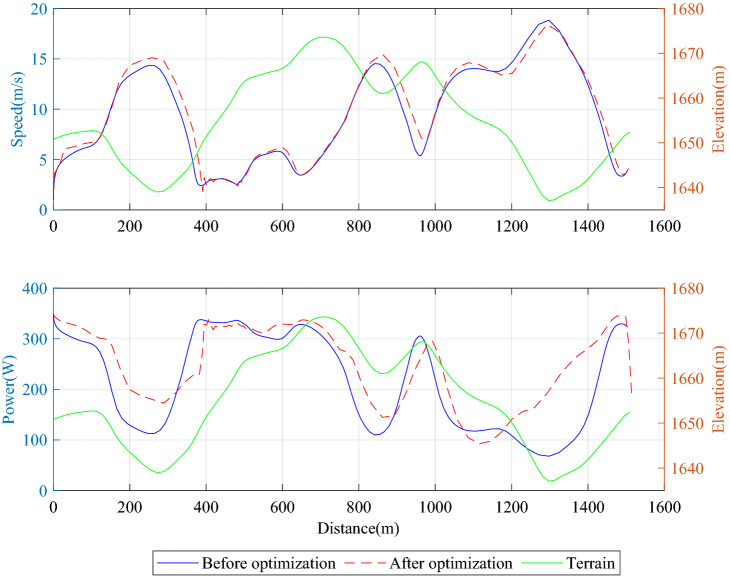
Global speed and driving power distribution of athlete A before and after optimization.

From the comparison of the speed and power distribution of athlete A before and after optimization shown in Fig. 10, it can be seen that a more conservative pace strategy is adopted in the uphill section of 350–500 m to save physical strength; the flat area of 500–600 m maintains a power output that is 40 W higher than before optimization, it will not increase fatigue due to excessive power changes; increase power output in the first downhill section of 700–850 m in the second half to win the initial speed for the subsequent uphill sections; in the final segment, all the energy saved will be output and the total power sprint.

Therefore, in long uphill, one should try to save physical strength based on the existing strategy; in long-distance downhill, it is necessary to use the terrain to accelerate as much as possible to get a higher initial speed for the next uphill segment. At the same time, this also puts higher requirements for the athletes' skiing stability; in the final segment of the journey, they must sprint with all their strength.

It is worth mentioning that the average power is not constrained in the optimization, and the athletes' average power output after optimization increases by 5.8% compared with that before optimization. Results from the study by Swarén et al.^18^ show that the male athlete's average power output increases by more than 8.9% in the time trail compared with that in the final, which means that the athletes' average power output is variable in the race and should not be considered as constant. In the study by Wolf et al.^19^ and Dahmen^20^ for the pacing strategy of road cycling, they also use the total energy output and the peak power as the constraints.

### Comparative analysis of pacing strategies between athlete A and champion athlete

Through the comparative analysis of the optimized simulation data of athlete A and the competition data of champion athletes, on the one hand, we can analyze the differences of pacing strategies between athletes of different levels; On the other hand, the difference of power and energy output can be compared to provide a reference for the subsequent formulation of the physical training program.

The physiological data such as height, weight, and maximum oxygen consumption of champion athletes are collected in the appendix. According to the relationship between maximum oxygen uptake and peak power proposed by Burnley and Vanhatalo^12^ and the power collection of champion athletes by Dahl et al.^21^, the power curve of male champion athletes is obtained.

According to the speed data of champion athletes, the last downhill section in the second half (990–1250 m) can slow down the attenuation of output power under the condition of the controllable body, grab the time gain of 2.8 s, and also enable the initial segment of the last uphill section (1300–1550 m) to obtain a higher initial speed, to fully accelerate to exhaust all physical fitness, It is verified that one of the critical points of optimization mentioned above: it is necessary to improve the initial speed of the next segment by taking advantage of the low cost of physical consumption caused by the downhill speed gain in the terrain connection section. In other words, athlete A cannot fully accelerate in the downhill, especially in the second half of the downhill, due to psychological and environmental factors, resulting in the decline of results. Therefore, one of the critical points of athlete A's speed-up strategy is to overcome the psychological obstacles of slowing downhill acceleration and improve physical control, which requires communication and negotiation between the coaching team and athletes.

**Figure 11:**
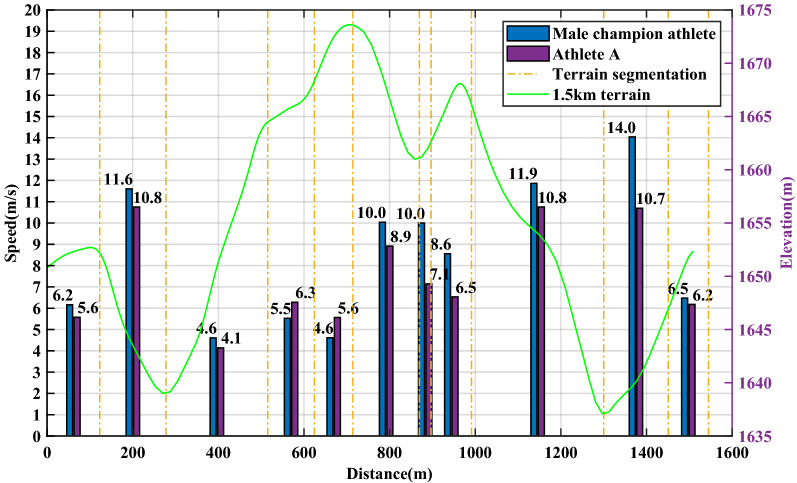
Speed distribution comparison of athlete A and male champion athlete.

**Figure 12:**
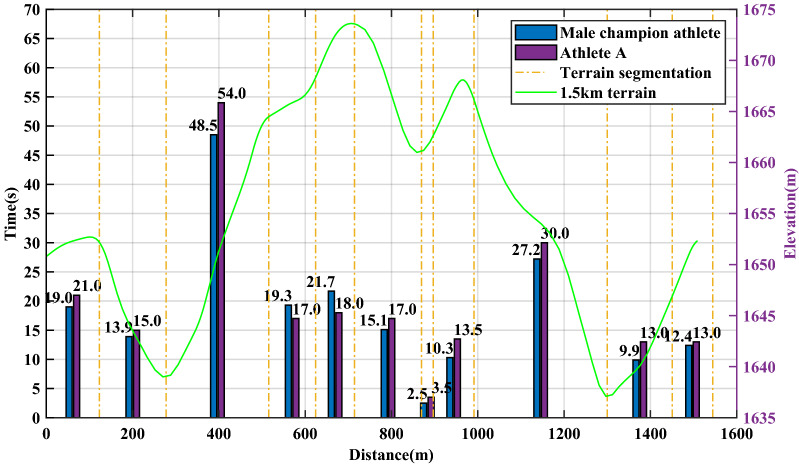
Time distribution comparison of athlete A and male champion athlete.

To more clearly observe the changes of pacing strategy of two athletes in the whole process, we can see from Figs. 11 and 12 that in the front section of the gentle uphill of 620–700 m, the front section of the downhill of 750–790 m and the front section of the downhill of 980–1030 m, compared with domestic athletes, foreign champion athletes started earlier, to obtain a higher initial speed in the downhill section.

**Figure 13:**
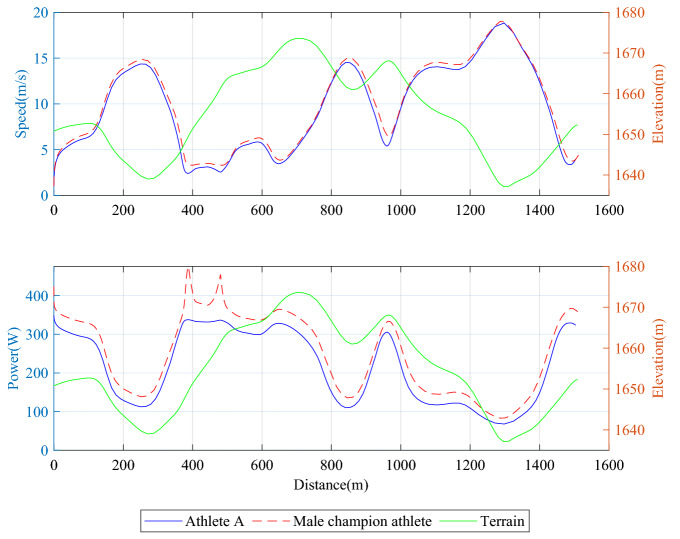
Global speed and driving power distribution of athlete A and champion athlete.

From the instantaneous power data of athletes shown in Fig. 13, the first uphill in the first half of the journey, especially for the slight change of the uphill slope with a slope of 0.08%–0.15%, the champion athletes have a keener awareness in adjusting their power output. Especially in the uphill section, the output power curve of the champion athletes is almost parallel to the change of terrain slope.

The following conclusions can be drawn by comparing the pacing behavior of domestic and foreign champion athletes. First, for the treatment of the first uphill, due to the solid physical reserves of champion athletes, they will choose to adopt more laborious uphill technology to maintain the slow decline of speed and ensure a sizeable initial speed in the climbing segment. Therefore, athlete A should appropriately increase the speed of the first uphill section according to the physical reserves. Secondly, for the downhill section, both champion athlete and athlete A have strategies to reduce the output power to slow down the rapid rise of speed. However, athlete A chose to reduce the output power earlier and reduced the output power more, resulting in speed lag. Therefore, athlete A should enhance the adaptability and stability of high-speed skiing in the downhill section and use the high-speed skiing in the downhill section to remedy the deficiency of complement energy.

In addition, the speed change trend of the optimized pacing strategy is basically the same as that of the champion athletes. It is closer to the performance of the champion athletes than that before the optimization, which can also further confirm the feasibility of the speed optimization method.

## Discussion

The results of this study confirm the results of Swain that major time savings can be realized by utilizing a variable pacing strategy. But different from the research results of Sundström et al., they believe that the optimization program strives to increase the propulsion power on the uphill and reduce the propulsion power on the downhill. However, it can be seen in Fig. 10 that the optimization results do not increase the propulsion power on all uphill sections. For example, in the uphill section with a gentle slope of 600–700 m, the propulsion power should be increased to slow down the loss of speed; However, in the uphill section with a sharp slope of 300–500 m, the propulsion power should be properly maintained or slightly reduced to preserve physical strength. On the other hand, the propulsion power should be increased in the subsequent downhill section to improve the sliding speed in the downhill section, to reduce the competition time, and provide a higher initial speed for next uphill section.

The difference in optimized strategies may be due to several aspects. Firstly, the optimization constraints are different. The differences in competition terrain result in significantly different pacing strategies. The terrain altitude range selected by Sundström et al. in their article is about 10 m, while the terrain fluctuation studied in this paper is larger, with an altitude change range of about 60 m. In specific applications, the pacing strategy should be designed separately for each track and each athlete to achieve the best results. Secondly, the different optimization algorithms may lead to different optimized strategies. Sundström et al. used the Method of Moving Asymptotes (MMA) to optimize the problem, while this paper chose the Gaussian pseudo-spectral method. These two algorithms are both highly reliable optimization algorithms, which should not have a great impact on the results.

Due to the terrain data in other articles is hard to be obtained, there is no quantitative comparative analysis yet. In the subsequent research, we can further explore the differences of the pacing strategies given by the optimization algorithms under different terrain conditions, as well as the optimization results of different optimization algorithms under the same terrain data.

The method in this paper is not perfect, for example, only the total time, energy output, and power output range are considered in the constraint conditions of the optimization problem, and the maximum power duration, different skiing skills and other factors that may have important influence on the results are not considered, so further research will be carried out in the subsequent work.

## Conclusion

Aiming at the development trend of scientific training, this paper introduces the kinematics, and dynamics model of cross-country skiing reproduces the motion process through numerical simulation; combined with the Gauss pseudo-spectral method, the optimization framework of cross-country skiing pacing strategy is established.

In this paper, the feasibility of using numerical simulation and optimization methods to optimize the pacing strategy behavior of cross-country skiing is demonstrated. The research results of pace optimization show that although the physical cost of accelerating on the downhill is lower than other section, however, athletes are usually not fully accelerated due to psychological, environmental, training or other factors, leaving room for further improvement on performance, even for top athletes. Therefore, if athletes focus on training acceleration strategies and skills in the downhill process, better results could be achieved.

Based on the measured data of Chinese athlete A, the original pacing strategy is optimized under the constraint of constant total energy output. The optimized pacing strategy can be summarized that in the first significant uphill section, a recommendation to athlete A is to use a more conservative pacing strategy, while in the latter half of the uphill section the power output should be increased, and maintain the power output in downhill sections. In the flat track section the power output should be as high as possible within the capacity.

The current results are preliminary, however, there are significant challenges remaining to be addressed. For example, the optimization algorithm based on the Gaussian pseudo-spectral method can be further improved; the multi-rigid body dynamics can be applied to the motion model; especially, the optimization results can be really applied to the actual competition. If there is an athlete test verification, the feasibility of the optimized pace strategy can be further verified, but limited by the actual situation, the optimization analysis of the pacing strategy is only analyzed from the theoretical level (Supplementary Information).

## Supplementary Information

Supplementary Tables.
