# Author Correction: Numerical optimization of pacing strategy in cross-country skiing based on Gauss pseudo-spectral method

**DOI:** 10.1038/s41598-022-26068-3

**Published:** 2022-12-14

**Authors:** Xu Ni, Jiawei Liu, Shuguang Zhang, Peng Ke

**Affiliations:** School of Transportation Science and Engineering, Beihang University, Beijing, 100191 China

Correction to: *Scientific Reports* 10.1038/s41598-022-24859-2, published online 28 November 2022

The original version of this Article contained an error in the legend of Figure 5. The authors omitted the below Reference, which is listed as Reference 15.

15. Reid, R. C. A kinematic and kinetic study of alpine skiing technique in slalom. Norwegian School of Sport Science. (2010).

The legend of Figure 5:

“Force analysis diagram.”

now reads:

“Force analysis diagram^15^.”

As a result, in the Pacing strategy optimization method section,

“As shown in Fig. 5, when the initial conditions are known, the dynamic trajectory planning problem can be transformed into a series of static optimization problems ^13,15^; in fact, this process is to transform the two-point boundary value problem corresponding to the continuous first-order necessary condition of the original optimization problem into an initial value problem^16^.”

now reads:

“As shown in Fig. 5^15^, when the initial conditions are known, the dynamic trajectory planning problem can be transformed into a series of static optimization problems ^13,16^; in fact, this process is to transform the two-point boundary value problem corresponding to the continuous first-order necessary condition of the original optimization problem into an initial value problem^17^.”

As a result of the changes, the References have been renumbered.

The original Article has been corrected.

