# Supplementary material for: Numerical optimization of pacing strategy in cross-country skiing based on Gauss pseudo-spectral method
Source: Sci Rep. 2022 Nov 28;12:20457. doi: 10.1038/s41598-022-24859-2 (PMC9705566; doi:10.1038/s41598-022-24859-2)
Supplement: Supplementary file 1 — Supplementary Tables. [file 41598_2022_24859_MOESM1_ESM.docx]

# Appendix

**Table 1** Parameters

| **Parameter** | **Unit** | **Physical meaning** |
| --- | --- | --- |
| $P_{th}$ | $W$ | The peak value of power output, determined by the weight, metabolic rate and lactate threshold of skiers |
| $\alpha$ | $s/m$ | The $\alpha$ the smaller, the lower the rate at which the output power decreases with the increase of speed |
| $b$ | ${rad}^{-1}$ | The higher the $b$, the higher the rate at which the output power rises with the increase of terrain slope |
| $c$ |  | Other factors affecting power output except for speed and slope |
| $\alpha_{1}$ | ° | Vertical terrain slope angle |
| $\alpha_{2}$ | ° | Lateral terrain slope angle |
| $\gamma$ | ° | The included angle between speed direction and X-axis of track coordinate system |
| $\beta$ | ° | Ski tilt angle |
| $F_{f}$ | $N$ | Frictional resistance |
| $F_{D}$ | $N$ | Aerodynamic resistance |
| $F_{N}$ | $N$ | Ground support force |
| $v$ | $m/s$ | Skiing velocity |

Table 2 Physiological data of athlete A and champion athlete

| **Athlete** | **Gender** | **biometric data** | | | **Energy output data** | |  |
| --- | --- | --- | --- | --- | --- | --- | --- |
|  |  | Height （cm） | Weight（kg） | Body fat ratio（%） | Maximum oxygen uptake（L/min） | Blood lactic acid（mmol/L） | |
| A | Male | 176 | 67 | 14.2 | 4.75±0.36 | 2.2±0.7 | |
| Champion | Male | 182 | 76 | 13 | 4.88±0.40 | 2.7±0.7 | |

Table 3 Driving power model related data of athlete A and champion athlete

| **Athlete** | $\boldsymbol{P}_{\boldsymbol{th}}\mathbf{(W)}$ | $\boldsymbol{\alpha(}\mathbf{s/m}\boldsymbol{)}$ | $\boldsymbol{b(}\mathbf{rad}^{\mathbf{-1}}\boldsymbol{)}$ | $\boldsymbol{c}$ |
| --- | --- | --- | --- | --- |
| A | 323±27 | 5.3±1 | 20±3 | Depends on current power output |
| Champion | 442±30 | 4±2 | 27±6.5 | Depends on current power output |
